# The Anatomical Differences and Physiological Responses of Sunburned Satsuma Mandarin (*Citrus unshiu* Marc.) Fruits

**DOI:** 10.3390/plants11141801

**Published:** 2022-07-07

**Authors:** Misun Kim, Yosup Park, Seok Kyu Yun, Sang Suk Kim, Jaeho Joa, Young-Eel Moon, Gyung-Ran Do

**Affiliations:** 1 Citrus Research Institute, National Institute of Horticultural & Herbal Science, Rural Development Administration, Seogwipo-si 63607, Jeju-do, Korea; mkim2019@korea.kr (M.K.); sky0611@korea.kr (S.K.Y.); sskim0626@korea.kr (S.S.K.); choa0313@korea.kr (J.J.); yimoon@korea.kr (Y.-E.M.); 2 Planning and Coordination Division, National Institute of Horticultural & Herbal Science, Rural Development Administration, Wanju-gun 55365, Jeollabuk-do, Korea; microdo@korea.kr

**Keywords:** chlorophyll, flavonoids, reactive oxygen species, satsuma mandarin, sunburn

## Abstract

Sunburn causes fruit browning and other physiological symptoms, reducing fruit production and quality. Therefore, we aimed to investigate the anatomical differences and abiotic stress responses in ‘Nichinan 1 gou’ satsuma mandarin (*Citrus unshiu* Marc.) according to the severity of sunburn damage (five grades: control, no sunburn; I to IV, increasing severity of sunburn). Additionally, the quality of sunburned and non-sunburned fruits was compared, and the sunburn-inducing temperature was estimated. Anatomical observations confirmed that with increased severity of symptoms, the damage to fruit rind surface and oil glands was increased. In the analysis of peel pigments, chlorophyll content in the rind gradually decreased compared with IV, whereas the carotenoid content gradually increased up to III. The flavonoid content in the peel and pulp was the highest in III. In the 1,1-diphenyl-2-picrylhydrazyl and 2,2′-azinobis (3-ethylbenzothiazoline-6-sulfonic acid) radical analyses, the IC50 (the concentration of compound at which the percentage of inhibition is 50%) value was the lowest in grade III in peel or IV in pulp, indicating a high free radical scavenging ability. The fruit quality analysis between sunburned and non-sunburned fruits showed differences in total soluble solid content, total acidity, firmness, coloration, and free sugar and organic acid contents, indicating a significant effect on fruit quality. In the heat tolerance tests on fruit rind in the laboratory and field, the damage was confirmed at temperatures above 47 °C.

## 1. Introduction

High temperatures in summer have a dramatic effect on crop growth and quality. Generally, leaf wilting, fruit cracking, and sunburn symptoms are physiological disorders that lower the production and quality of fruits. Sunburn, due to rapid temperature rise, causes browning of fruit surfaces and fruit drop. Sunburn has been reported in fruit crops, such as apples, tomatoes, and grapes [1,2,3,4]. In apples, Racsko and Schrader [5] noted that sunburn is caused by direct factors, such as excessive radiant heating and/or daylight and indirect factors, which influence the appearance and severity of the symptoms.

Global warming has increased surface temperature worldwide 0.99 °C from 1850 to 1900 to 2001 to 2020 and by 1.09 °C from 1850 to 1900, to 2011 to 2020, and this increase is still in progress [6]. The increase in the average temperature and frequency of heat waves in summer has been evident since the mid-1990s and is common in Korea and East Asia [7]. Park et al. [8] reported that the sunburn risk days in 2010–2019 were more than twice the number in previous decades. Therefore, it is expected that fruit damage from sunburn will continue to increase in the future.

Citrus fruits contain various physiologically active substances with phenolic compounds, vitamin C, flavonoids, and carotenoids, indicating beneficial health-related properties, such as anti-cancer and anti-inflammation [9,10,11]. Citrus is highly valued as raw fruit and a food additive using its by-products [12]. In unripe citrus fruits, the content of polyphenols and flavonoids is higher than in mature fruits [13].

Among the citrus species, satsuma mandarins (*Citrus unshiu* Marc.) accounted for more than 87% of domestic citrus production (approximately 650,000 metric tons) in 2020 in South Korea [14]. ‘Nichinan 1 gou’ satsuma mandarin (*C. unshiu* Marc.), the most cultivated cultivar among the very early-ripening cultivars (mature in late September to October), is a bud mutation of the early-ripening cultivar ‘Okitsu wase’ satsuma mandarin (*C. unshiu* Marc.). The susceptibility to sunburn differs according to the citrus cultivars [15,16]. Sunburn is more severe in the early-ripening cultivars, significantly decreasing early citrus production [8]. Particularly, it is difficult to distinguish between sunburned and non-sunburned fruits when the fruit skin starts to color after the high-temperature period in summer. Additionally, unripe satsuma mandarin passed 85 to 130 days after full bloom (DAFB) during the hot summer, is used as a functional processed product, and their production reached 950 metric tons in 2019 [17]. Therefore, sunburned satsuma mandarin fruits are undesirable among farmers and consumers. Although previous citrus studies to suppress sunburn using kaolin, calcium carbonate, and shading treatment have been conducted [18,19,20], few systematic studies showed the effect of sunburn on anatomical difference and physiological responses to satsuma mandarin quality.

Reactive oxygen species (ROS) levels are widely used for monitoring citrus environmental stress [16,21,22], and an increase in ROS leads to pigment destruction, lipid peroxidation, cell membrane damage, and cell necrosis [23,24].

In apples, sunburn browning appears at increased fruit surface temperatures (FST) of 46–49 °C, whereas sunburn necrosis occurs at 52 °C [3]. In ‘Miyagawa wase’ (C. *unshiu* Marc) satsuma mandarin, sunburn was induced at 45 °C through heat treatment [25], while Park et al. [8] suggest that predictions of sunburn occurred above 46 °C in ‘Nichinan 1 gou’ satsuma mandarin fruit by prediction modeling. However, studies on the heat tolerance of rind closely related to sunburn in ‘Nichinan 1 gou’ satsuma mandarin are insufficient.

The study aimed to investigate the anatomical differences and abiotic stress responses in ‘Nichinan 1 gou’ satsuma mandarin according to the severity of sunburn. Furthermore, we determined the temperature at which heat tolerance in rind occurs, as this data could be used for designing strategies to prevent the damage caused by sunburn.

## 2. Results

### 2.1. Experimental Conditions

**Figure 1:**
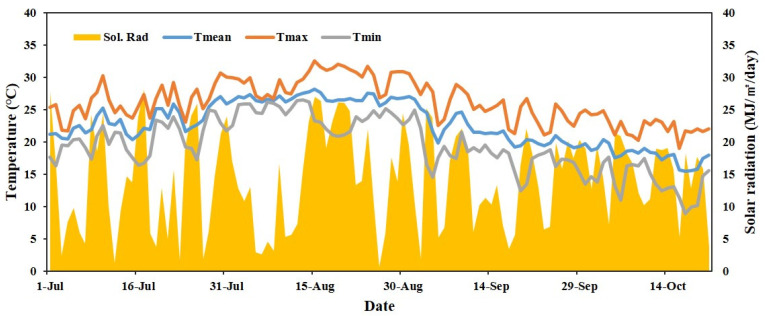
Daily average of maximum (Tmax), mean (Tmean), and minimum (Tmin) temperatures and solar radiation (Sol. Rad.) from July to October 2020.

The daily average temperature and solar radiation throughout the experimental duration are indicated in Figure 1. The daily average maximum temperature and solar radiation in July was 30.7 °C and 21.2 MJ/m^2^/day, respectively. In August, the daily average maximum temperature and solar radiation was 32.6 °C and 26.1 MJ/m^2^/day, respectively.

### 2.2. Histological Observations according to the Sunburn Damage Severity

**Figure 2:**
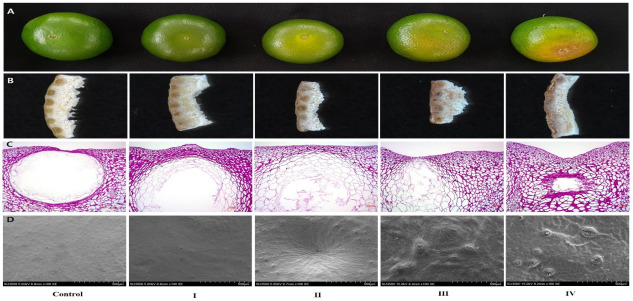
Anatomical and histological observations of unripe Satsuma mandarin fruits. (**A**) Unripe Satsuma mandarin fruits with different degrees of sunburn: non-sunburned (control), slight (I), mild (II), moderate-severe (III), and severe (IV); (**B**) Stereoscopic and (**C**) light microscopic images of the longitudinal section of fruit rind showing the flavedo and albedo layers. (**D**) Scanning electron microscope (SEM) images of the cuticular wax on the surface of unripe Satsuma mandarin fruits.

The surface and cross-section of the peel were observed for sunburn symptoms using an optical microscope and a scanning electron microscope (SEM) (Figure 2). Based on the tissue observations using an SEM, cracks on the oil gland wall of the flavedo layer were confirmed to form, resulting gradually in shrinkage as the symptoms deteriorated. Additionally, the cuticle—the peel surface—became rough and cracked and eventually underwent necrosis and pathogen invasion.

### 2.3. Physiological Analysis

#### 2.3.1. Pigment Analysis

**Figure 3:**
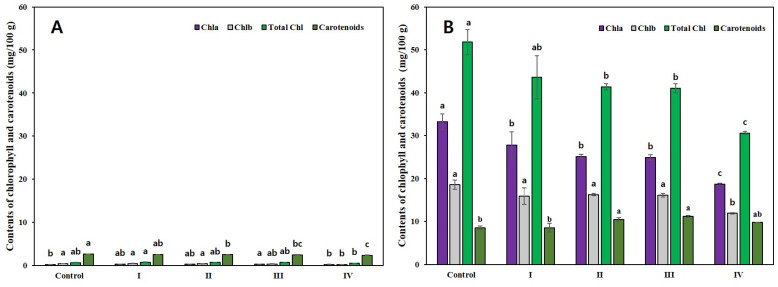
Chlorophyll and carotenoid contents (mg/100 g dry weight) in Satsuma mandarin fruit (**A**) pulp and (**B**) peel, according to the degree of sunburn severity. The values representing different alphabets are statistically significant (*p* < 0.05).

The changes in chlorophyll and total carotenoid contents of the pulp and peel were analyzed at varying severities of sunburn: non-sunburned (control), slight (I), mild (II), moderate-severe (III), and severe (IV). The results indicated that the chlorophyll and total carotenoid content of the pulp were lower than that of the peel (Figure 3). In the pulp, the chlorophyll *a* content was the highest in III, and the chlorophyll *b* and total chlorophyll content were the lowest in IV.

In the peel, the contents of chlorophyll *a*, *b*, and total chlorophyll in the control were 33.2, 18.5, and 51.9 mg/100 g, respectively, and showed a gradual reduction as the sunburn symptoms deteriorated. The total carotenoid content in the pulp of the sunburned fruits decreased as the symptoms deteriorated and were lower than that of the control. In contrast, the carotenoid content in the peel was the lowest in the control and the highest in III.

#### 2.3.2. Flavonoid Analysis

The levels of phenolic compounds are related to various physiological characteristics, including antioxidant and antibacterial characteristics. Among > 60 types of flavonoids, the main flavonoids identified according to citrus cultivars are diverse, and the flavonoids in the peel are abundant [26,27,28]. Among the ten major flavonoids analyzed, seven flavonoids were identified (Table 1). However, naringin, neohesperidin, and nobiletin were not detected in the pulp.

In the pulp, the rutin content was doubled in III compared to those in the control. Moreover, the narirutin content increased approximately 1.8-fold in III compared to that in the control. However, the hesperidin content increased approximately 1.4 times in IV compared to that of the control. The total flavonoid content increased approximately 1.6 times in III compared to that of the control.

In the peel, the rutin content increased approximately 2.1 times in III compared to that of the control. Additionally, the narirutin content increased 1.1 times in III compared to that of the control. The naringin content gradually decreased with the severity of symptoms. The nobiletin and tangeretin contents were lower than those of the control; however, the difference was not significant. The total flavonoid content increased approximately 1.1 times in III compared to that of the control.

#### 2.3.3. Malondialdehyde (MDA) Analysis

**Figure 4:**
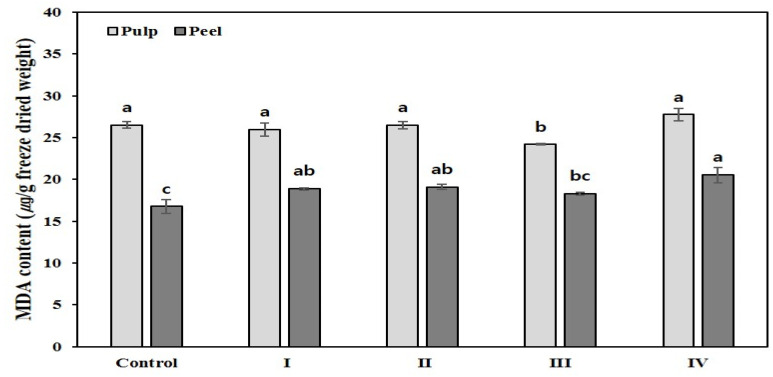
Malondialdehyde (MDA) contents (μg/g freeze-dried weight) according to the degree of sunburn in fruits of Satsuma mandarin. The values representing different letters are statistically significant (*p* < 0.05).

The MDA analysis was used to determine the oxidative lipid product of stress-induced tissue damage. In the pulp, the MDA content in III was slightly lower than that in the control; however, there was no difference in the other severities. In contrast, in the peel, the MDA contents in I–IV increased by approximately 1.1 to 1.2 times compared to that of the control (Figure 4).

#### 2.3.4. Total Phenolic Content (TPC), Ferric Reducing Antioxidant Power (FRAP), and Antioxidant Activities

**Figure 5:**
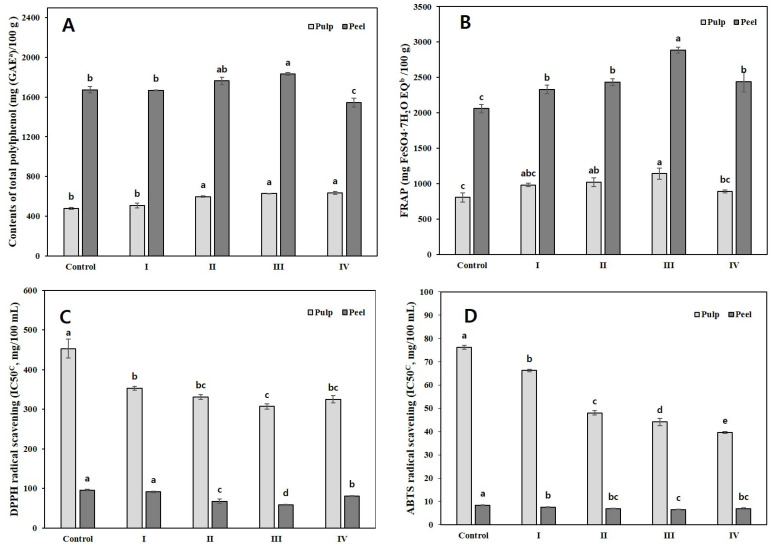
Total polyphenol contents and antioxidant activities according to the degree of sunburn in Satsuma mandarin fruits. (**A**) Total polyphenol contents (TPC); (**B**) Ferric reducing antioxidant power (FRAP); (**C**) 1,1-diphenyl-2-picrylhydrazyl (DPPH) radical scavenging activity; (**D**) 2,2′-azinobis (3-ethylbenzothiazoline-6-sulfonic acid (ABTS) radical scavenging activity. ^a^ TPC values are expressed as mg of gallic acid equivalents (GAE) per 100 g of 70% ethanol extracts. ^b^ FRAP values are expressed as mg of Iron (II) sulfate heptahydrate (F_2_SO_4__·_7H_2_O) equivalents per 100 g of 70% ethanol extracts. ^c^ IC50, concentration in mg per 100 mL required for scavenging the DPPH and ABTS radicals by 50%. The data are expressed as mean ± SE (*n* = 3). The values representing different letters are statistically significant (*p* < 0.05).

The total polyphenol content (TPA) of fruits with varying severity of sunburn is shown in Figure 5A. In the pulp, the TPA in grade III was 627.6 mg/100 g, whereas the TPA in the control was 480.0 mg/100 g, indicating a noticeable increase in severity. Similarly, in the peel, the TPA increased as the severity increased. The TPA in III was 1831.4 mg/100 g, which was approximately 1.1 times higher than that of the control; however, the TPA decreased rapidly to 1544.0 mg/100 g in IV.

The total antioxidant capacity, measured using the FRAP method, showed that the antioxidant capacity of the pulp and peel increased gradually with the severity of the symptoms (Figure 5B). The pulp and peel showed the highest total antioxidant capacity in III with 1141.0 mg/100 g and 2886.8 mg /100 g, respectively.

Figure 5C shows the result of the 1,1-diphenyl-2-picrylhydrazyl (DPPH) radical scavenging assay. The IC50 (the concentration of compound at which the percentage of inhibition is 50%) value in the pulp of DPPH radical scavenging activity decreased approximately 1.3 times in III compared to that of the control. Similarly, the IC50 value of the peel decreased approximately 1.6 times in III compared to that of the control. The IC50 value of 2,2′-azinobis (3-ethylbenzothiazoline-6-sulfonic acid) (ABTS) also showed a similar trend to that of DPPH radical scavenging activity (Figure 5D). The IC50 value of the pulp decreased approximately 1.9 times in IV compared to that of the control. Moreover, the IC50 value in the peel was decreased approximately 1.3 times in III compared to that of the control.

#### 2.3.5. Analysis of Fruit Quality and Free Sugar and Organic Acid Contents

Table 2 shows the results of the total soluble solids (TSS) content, acidity, firmness, and color, which indicate a significant difference between sunburned and non-sunburned fruits. At harvest, the TSS contents of sunburned and non-sunburned fruits were 8.6 °Brix and 9.0 °Brix, respectively. Moreover, the acid content was low in sunburned fruits. However, the firmness of the sunburned fruits increased approximately 1.6 times compared to that of the non-sunburned fruits, indicating a considerably hardened peel. The brightness value of the non-sunburned fruits was higher than that of the sunburned fruits. The positive b* value, indicating yellow chromas, was also higher in the non-sunburned fruit. However, there was no difference in the positive a* value, indicating red chromas.

**Figure 6:**
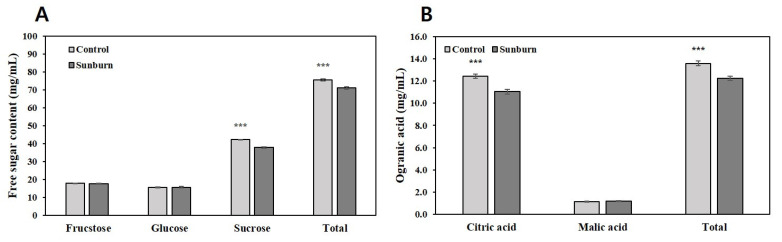
Comparison of free sugar and organic acid contents in sunburned and non-sunburned (control) Satsuma mandarin fruits. The values represent mean ± SE of 30 replicates. *** *p* < 0.001 (Student’s *t*-test).

Based on the free sugar analysis (Figure 6A), the fructose and glucose contents of the non-sunburned fruit were 17.9 mg/mL and 15.6 mg/mL, respectively, and there was no difference in the contents compared with those of the sunburned fruit. However, the sucrose content of the sunburned fruits decreased approximately 0.9-fold compared to that of the non-sunburned fruits. In the organic acid analysis (Figure 6B), the citric acid content of the sunburned fruit decreased approximately 0.9 times compared to that of the non-sunburned fruits. There was no difference in the malic acid contents between the sunburned and non-sunburned fruits.

#### 2.3.6. Heat Tolerance and Evaluation of Cell Membrane Damage

**Figure 7:**
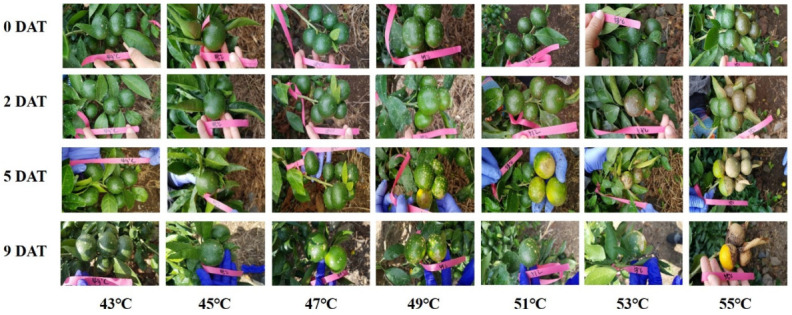
Heat tolerance induced by soaking treatments at different water temperatures. White or brown spots indicate the areas where sunburn-like symptoms were observed.

The heat tolerance occurrence temperatures were induced through high-temperature immersion treatments at 43–55 °C. Browning of the fruit surface was observed at temperatures above 53 °C. As the treatment period elapsed, discoloration was gradually centered on the oil glands at temperatures above 47 °C (Figure 7).

**Figure 8:**
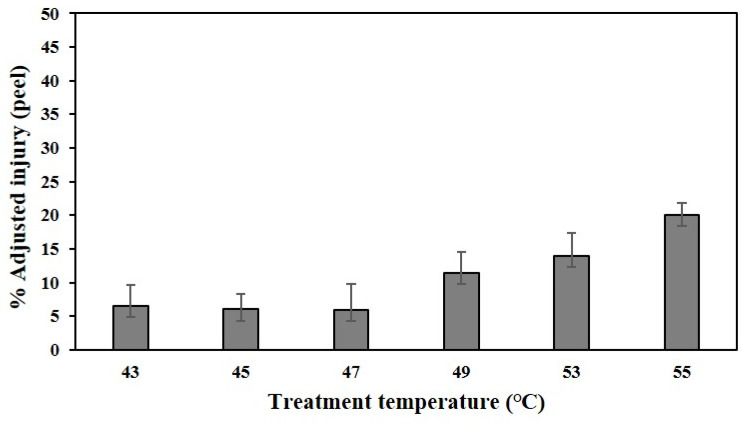
The percentage of adjusted injury on peels of Satsuma mandarin fruits subjected to soaking treatments at different water temperatures. The data are expressed as mean ± SE (*n* = 5).

Electrolyte leakage (EL) is mainly used to evaluate the degree of cell breakdown following cell membrane damage [29]. High-temperature stress increased cell membrane permeability. Figure 8 shows the results of the degree of cell disintegration of citrus peels after varying high-temperature water immersions. The percentage of adjusted injury increased rapidly at temperatures above 49 °C.

## 3. Discussion

Citrus fruit rind (peel) comprises a flavedo layer containing oil glands and pigments, and an albedo layer—a white, spongy region containing pectin and soluble fibers. The cuticle, which is the outermost region of the flavedo layer, not only plays a critical role in the development and postharvest of citrus fruit but also serves as an important barrier between the fruit and the environment. Moreover, it determines the gas exchange rate of respiratory metabolites and flavor volatiles and protects against insects and microorganisms [30,31]. The inner flavedo cells and oil glands increase in size during fruit development [32]. Based on the anatomical analysis shown in Figure 2, the oil gland of the flavedo layer collapsed (II to IV), and the cuticle cracked gradually, depending on the severity of the symptoms. Moreover, the invasion of external pathogens (III and IV) was confirmed.

These results suggested that the flavdeo layer, which was exposed directly to external environmental stresses, such as high temperatures and intense solar radiation, was more susceptible to damage than the albedo layer, significantly inhibiting the barrier function.

The loss of chlorophyll in the citrus peel nearly corresponded to the onset of carotenoid accumulation [33]. Based on the sunburn symptoms, the chlorophyll and total carotenoid contents in the pulp were significantly lower than those in the peel (Figure 3). This was because the pigment layer was mainly present in the peel.

The total chlorophyll content in the pulp and peel decreased with the severity of symptoms. In contrast, the total carotenoid content in the peel continued to increase until III, which was observed before peel hardening. Similarly, in the Fuji apple cultivar, the chlorophyll *a*, *b*, and anthocyanin content were lower in the sunburned peel than in the non-sunburned peel. Meanwhile, the content of chlorogenic acid, beta-carotene, xanthophyll pigments, and major quercetin glycosides was higher in the sunburned peel than in the non-sunburned peel [34].

Chlorophyll is a photosynthetic pigment that collects light energy and induces electron transfer. Decomposition of chlorophyll plays a role in protecting plants from phototoxic pigments [35,36]. Hu et al. [37] reported that under heat stress, the levels of chlorophyllase and chlorophyll degrading peroxidase increased, while the chlorophyll content decreased significantly.

Therefore, in the ‘Nichinan 1 gou’ mandarin fruits, the increase in the total carotenoid content seemed to protect against damage from external stress, such as sunburn. However, the beta-carotene content increased or decreased in sunburned apple fruits depending on the cultivar [34,38,39]. Felicetti and Schrader [40] explained that apples showed a significant difference in the beta-carotene content according to the sensitivity of cultivars to sunburn. This suggested that the change in the total carotenoid content within the sunburned fruit might vary depending on the citrus cultivar.

Among the flavonoids, narirutin and hesperidin were most abundantly detected in ‘Nichinan 1 gou’ mandarin fruits (Table 1). In previous studies, the two most abundant flavanones were seen in significant amounts regardless of citrus cultivars or their different organs, such as a leaf, peel, and pulp [13,41,42]. According to the previous study [13,43], flavonoid content in satsuma mandarin fruits tended to decrease as the fruit matured. Flavonoids, along with phenolic compounds, play an essential role in protecting plants from ultraviolet radiation (UV; 230–380 nm) [44]. Flavonoids are known to accumulate mainly in vacuoles of epidermal cells after exposure to UV [45]. In our study, the total flavonoid contents of the pulp and peel were the highest in III. Therefore, the flavonoid accumulation is due to sunburn stress because it was shown that the mechanism of normal fruit development is different.

The MDA content of the sunburned peels (Figure 4), which could confirm the degree of lipid change due to stress, was slightly higher than that of the control. Particularly, it was the highest in IV, in which the peel appeared to firm up. Similarly, Wan et al. [16] found that superoxide anion (such as ROS) accumulation in the pericarp of sunburned fruits was significantly higher than that in non-sunburned fruits and was proportionate to the susceptibility of the cultivars to stress. It was also indicated that the lipoxygenase activity and MDA content, which indicate deterioration of cell membrane and lipid peroxidation, were significantly increased.

The correlation between TPA and antioxidant capacities, such as DPPH or FRAP reported a significant difference in citrus fruits [46,47]. The TPA and FRAP were also the highest in III; however, the result of ROS scavenging activity, and the IC50 value of DPPH, were the lowest in III for both the peel and pulp. The IC50 value of ABTS was the lowest in III for the peel and IV for the pulp, indicating a high free radical scavenging activity (Figure 5). It has been reported that the contents of total flavonoids, rutin, hesperidin, tangeretin, and total polyphenols have a significant negative correlation with the IC50 value for DPPH and ABTS scavenging activities under citrus freezing stress [22]. Chen et al. [38] reported that the sunburned peel of apple showed a higher accumulation of H_2_O_2_ and MDA and an increased activity of antioxidant enzymes than the non-sunburned peel. Based on the observations, they inferred that the antioxidant system could not cope with the photooxidation induced by high temperatures combined with high light intensity. Lim et al. [48] proposed that macromolecule degradation and nutrient salvage and translocation, such as the loss of chlorophyll and nitrogen and lipid mobilization, occurred in response to various internal and external signals. Antioxidant and defense-related genes were activated, resulting in cell death.

Based on the observations of the present study, the fruits of ‘Nichinan No. 1’ fruits lost chlorophyll in response to the signal of external stress (sunburn) started in grade I, resulting in slight browning of the rind. To overcome the stress, the contents of defense-related carotenoids, antioxidants, and flavonoids and TPA were increased, and these appeared to have peaked in III, right before the peel hardening.

The results of the fruit quality analysis showed that at harvest time, the sugar and acid contents of sunburned fruits were significantly lower than those of non-sunburned fruits (Table 2). Additionally, the rind of sunburned fruits was considerably harder than that of the non-sunburned fruits, and the coloration was poor. Thus, sunburn is a factor that significantly lowers fruit quality. In contrast, as the degree of sunburn browning in apples increased, the hardness and TSS content increased, while the acid content decreased [34]. Scharder et al. [34] suggested that a significant decrease in water content with the increasing severity of sunburn could increase the sugar content. Unlike apples, citrus fruits are thought to have less water evaporation as the peel protects the flesh with two thick layers. However, it seems that further research on the topic is required.

According to Minessy et al. [15], the average annual temperature of citrus peels exposed to direct sunlight in summer is approximately 5–7 °C higher than the maximum atmospheric temperature. Park et al. [8] reported that the sunburn prediction modeling of ‘Ilnam No. 1’ fruit, using a temperature gradient tunnel, showed sunburn symptoms when the peel temperature threshold was 46 °C for more than 3 h.

In the present study, the temperature at which heat tolerance occurred in ‘Nichinan No. 1’ fruits was inferred through high-temperature water immersion in the field, and symptoms similar to sunburn were observed in the peel nine days after treatment at 47 °C (Figure 7). Moreover, at temperatures above 49 °C, the percentage of adjusted injury increased rapidly on the day of treatment (Figure 8). The electrical conductivity of peels damaged by photooxidative sunburn was approximately twice that of the undamaged peel [49]. This means that the membrane integrity was destroyed, increasing the EL. Therefore, the sun damage temperature threshold of ‘Nichinan No. 1’ fruits is approximately 47 °C. This result supports the peel temperature threshold suggested by Park et al. [8]. Therefore, the soaking treatment method appears to help test the heat tolerance of other cultivars or newly developed ones. Meanwhile, Park et al. [8] also suggested that solar radiation over a certain level had a relatively similar effect on the increase in peel temperature. Moreover, based on the multiple regression model analysis for peel temperature, air temperature, and solar radiation, it was found that solar radiation had a stronger (approximately 1.5 times) effect than the ambient temperature on peel temperature increase. Additionally, some studies reported that the tolerance to heat decreases according to the growth of the fruit, and the number of sunburns [15,25]. Further studies in these parts are likely to be necessary.

## 4. Materials and Methods

### 4.1. Plant Materials and Artificial Sunburn Induction

Eighteen-year-old ‘Nichinan 1 gou’ mandarin trees (planted at 2.7 m × 2.7 m spacings) grown in a very dark brown volcanic ash soil in the open field at the Citrus Research Institute of the Rural Development Administration (RDA) in Seogwipo-si, Jeju-do, Korea (33°18′06″ N; 126°36′39″ E), were used for this study. Fertilizer and pest control was performed according to the standard citrus cultivation method of the RDA. The rainfall from June to September by the automatic weather system (AWS) device at the experimental site was recorded at approximately 1800 mm.

For the comparison of histological and abiotic stress responses of fruits to natural sunburn based on the severity of sunburn: non-sunburned (control), slight (I), mild (II), moderate-severe (III), and severe (IV) fruits were randomly collected at 1.2–1.5 m above the ground on trees at 120 DAFB. The fruit with an approximately transverse diameter of 56 mm and longitudinal diameter of 48 mm was tested. To compare fruit characteristics, non-sunburned and sunburned fruits were investigated from 165 DAFB, which was the harvest time for the cultivar. Fruits were collected from each sample of 30 fruits.

To evaluate the artificial sunburn symptoms induced by a soaking treatment, fruits were tested at different water temperatures of 43, 45, 47, 49, 51, 53, or 55 °C for 10 min while still attached to the tree at 90 DAFB in early August. Three replicates were conducted in each treatment (5–7 fruit per replicate). To evaluate the degree of peel cell destruction, peel discs (thickness around 2.5 mm, 6.5 mm diameter) were cut out using a handheld one-hole paper punch, immersed in 20 mL of 12% sucrose solution, and then incubated in a water bath (SB-1200; Eyela Co. Ltd., Shanghai, China) at 25 (control), 43, 45, 47, 49, 53, 55, or 100 °C for 10 min. Five replicates were conducted in each treatment (13 peel discs per replicate).

### 4.2. Anatomical Analysis Using Light Microscopy and SEM 

The preconditioning process was a modification of the method described by Clément et al. [50]. Tissues were fixed in 2.5% glutaraldehyde solution (in a 100 mM phosphate buffer, pH 7.2) (EMS, Hatfield, PA, USA) in the presence of 4% sucrose (Sigma-Aldrich, Saint Louis, MO, USA) for 24 h. After three rinses (30 min each) with phosphate buffer, the samples were dehydrated in an alcohol series, transferred to propylene oxide, and embedded in historesin (Leica Microsystems, Wetzlar, Germany). The semi-thin sections (2.5 µm) prepared using an ultramicrotome (UC7, Leica Microsystmes) were collected on glass slides. The periodic acid–Schiff (PAS) polysaccharide-specific reaction was performed. The sections for staining were first plunged in 1% periodic acid (Sigma-Aldrich) for 30 min, and then in Schiff’s reagent for 40 min and finally in 5% sodium bisulfite (Sigma-Aldrich) for 35 min. The sections were then rinsed in distilled water, dried, and mounted in Histomount (EMS). The negative control was prepared by omitting the oxidation step with periodic acid and observed under a light microscope (Axioskop 2; Carl Zeiss AG, Oberkochen, Germany).

To examine the morphological characteristics and density of peel tissues, the tissues were fixed and dehydrated as described above, transferred to isoamyl acetate (EMS), and then dried in a critical-point dryer (HCP-2; Hitachi, Tokyo, Japan) with carbon dioxide as the intermediate fluid. The samples were then coated with gold-palladium for 1 min 30 s (100 Å) using an ion sputter (MC 1000; Hitachi) and examined using SEM (SU-3500; Hitachi) at an accelerating voltage of 15 kV.

### 4.3. Analysis of Pigment Contents 

The sunburned or non-sunburned fruits were collected, and the pulp and peel were separated and dried at 45 °C for 2 d until completely dehydrated. Each sample was ground to powder using a mini grinder. Then, 8 mL of 80% acetone was added to 400 mg powder, and the mixture was incubated in a shaking incubator at 25 °C for 24 h. The homogenate was centrifuged at 2700× *g* for 10 min at 25 °C. The absorbance of the supernatant was measured at 663.2 (662), 646.8 (645), and 470 nm using a UV-spectrophotometer (SpectraMax M2; Molecular Devices LLC., San Jose, CA, USA). The chlorophyll and carotenoid contents were calculated using a modification of the method described by Lichtenthaler [51].

Chlorophyll *a*, *b*, and total carotenoid contents were calculated using the following formula:

Chlorophyll *a* (μg/mL) = (12.25 × A662) − (2.79 × A645) (1)

Chlorophyll *b* (μg/mL) = (21.50 × A645) − (5.10 × A662) (2)

Total chlorophyll = chlorophyll *a* + chlorophyll *b* (3)

Total carotenoids (μg/mL) = (1000 × A470) − (1.8 × chl *a*) − (85.02 × chl *b*/198) (4)

where A662, A645, and A470 are the absorbance values at each wavelength, and chl *a* and chl *b* are the contents of chlorophyll *a* and chlorophyll *b*, respectively.

### 4.4. Lipid Peroxidation Analysis Using the MDA Assay 

The measurement of lipid peroxidation in the sunburned fruits was analyzed using the MDA assay according to the method described by Jakhar and Mukherjee [52]. The peels of the sunburned or non-sunburned fresh fruits were separated and frozen in liquid nitrogen. Fresh samples (50 mg) were ground into a powder using liquid nitrogen, mixed with 3 mL of 50 mM phosphate buffer (pH 7.0), and centrifuged at 15,000× *g* for 15 min at 4 °C. A one microliter aliquot of appropriately diluted sample was then added to a tube with 2 mL of 0.5% thiobarbituric acid in 20% trichloroacetic acid. All reagents used were obtained from Sigma-Aldrich (Saint Louis, MO, USA). The mixture was heated in a water bath for 30 min at 95 °C and then rapidly cooled in an ice bath. The samples were centrifuged at 10,000× *g* for 10 min at 4 °C. The absorbance of the supernatants was recorded at 532 nm and 600 nm using a UV spectrophotometer (UV-2700; Shimadzu Corp., Seoul, Korea). The concentration of MDA was calculated using an extinction coefficient of 155 mM^−1^ cm^−1^.

### 4.5. High-Performance Liquid Chromatography (HPLC) Analysis of Flavonoid Content and Antioxidant Activity

Sample extraction for the analysis of flavonoid content and antioxidant activity was conducted as described by Kim et al. [42]. The separated peel and pulp were thinly sliced, dehydrated at 45 °C for 2 days, and then ground to a powder for extraction. Approximately, 30 mL of 70% ethanol was added to 1 g of the powder, and the suspension was sonicated for approximately 1 h. The mixture was centrifuged at 3000× *g* for 10 min at 4 °C (Centrifuge 5810R; Eppendorf AG, Hamburg, Germany). The supernatants were passed through polytetrafluoroethylene (PTFE) syringe filters (0.45 μm) combined with a glass fiber (GF) pre-filter (Chromdisc, Taipei, Taiwan), and then centrifuged at 2700× *g* for 10 min at 25 °C. The final extracts were dried in a vacuum evaporator (HT-4X; Genevac Ltd., Suffolk, UK), dissolved in ethanol:dimethyl sulfoxide (1:1 *v*/*v*) (Supelco Inc., Bellefonte, PA, USA), and used for the measurement of flavonoid content and antioxidant activity.

The extraction solutions, 10-fold diluted with ethanol, were passed through a 0.2-μm polyvinylidene fluoride syringe filter. Flavonoids were analyzed using an HPLC system (e2695 Separations Module; Waters Corp., Milford, MA, USA) equipped with a UV/visible detector (Waters 2489, Waters Corp.) and separated on a YMC Korea–Triart C18 column (250 mm × 4.6 mm 5 μm; YMC Co., Ltd., Kyoto, Japan). The gradient conditions and mobile phase for HPLC were the same as those described by Kim et al. [13]. For quantification, a calibration curve was prepared using ten flavonoid standards (Sigma-Aldrich).

The TPC and antioxidant activities (DPPH and ABTS free radical scavenging assays] were determined using a spectrophotometer (SpectraMax M2; Molecular Devices LLC., San Jose, CA, USA). All assay conditions were as described by Kim et al. [42]. The TPA values are expressed as mg of gallic acid equivalents 100 g^−1^, and the 50% inhibitory concentration (IC50) is expressed as the quantity of the extract necessary to react with one-half of DPPH or ABTS radicals. The FRAP antioxidant activity analysis was conducted according to the method described by Kim et al. [13]. Briefly, 20 μL of extract solution diluted with ethanol was mixed with 180 μL of substrate solution in 300 mM sodium acetate buffer (pH 5.2): 10 mM 2,4,6-tripyridyl-s-triazine (in 40 mM HCl): 20 mM iron (III) chloride in distilled water in 10:1:1 (*v*/*v*/*v*) ratio. The mixed solution was incubated in the dark for 30 min at 25 °C, and then the absorbance values of the supernatant were measured at 590 nm. The concentration of FRAP was calculated based on a standard calibration curve prepared using iron (II) sulfate heptahydrate. All reagents used were obtained from Sigma-Aldrich.

### 4.6. Analysis of Fruit Properties

Sunburned and non-sunburned fruits (30 each) were randomly collected for the comparison of fruit characteristics in mid-October, which was the early-harvest time for the cultivar.

The CIELAB color space referred to as L*, a*, and b* parameters, were measured and averaged for three equatorial sites on the fruit surface using a CR-400 Chroma Meter (Konica Minolta Sensing Inc., Osaka, Japan). Fruit firmness was measured using a texture analyzer (TA-XT2; Stable Microsystem Ltd., Surrey, UK) fitted with a 3 mm (diameter) probe. The TSS content was determined using a refractometer (PAL-1, Atago Co. Ltd., Tokyo, Japan) after separating the pulp and juicing it. For acidic content analysis, the juiced samples were diluted five-fold with distilled water and titrated using a 0.1 N sodium hydroxide solution with one or two drops of 1% phenolphthalein solution. The juiced samples were stored at −80 °C and used for free sugar and organic acid analysis. All reagents used were obtained from Sigma-Aldrich.

### 4.7. HPLC Analysis of Sugar and Organic Acid

The juice was diluted 10 times with distilled water, and then 1 mL of each diluted extract was passed through PTFE syringe filters (0.2 μm) combined with a GF pre-filter (Chromdisc). The samples were analyzed using a UFLC Prominence system (Shimadzu Co., Ltd., Kyoto, Japan) equipped with a refractive index detector (RID 20A; Shimadzu Co., Ltd.) for analyzing sugars and a UV-Visible detector (SPD 20A; Shimadzu Co., Ltd.) for analyzing organic acids. For quantification, calibration curves prepared using sucrose, fructose, glucose, citric acid, malic acid, and oxalic acid standards (Sigma-Aldrich) were used. The HPLC operational and gradient conditions of the mobile phase for free sugar and organic acid analyses were the same as those described by Kim et al. [22].

### 4.8. Electrolyte Leakage Analyses

The EL was analyzed using a variation of the methods described by Dionisio-Sese and Tobita [53] and Scharader et al. [3]. Thirteen peel discs were incubated in 20 mL of 12 % sucrose solution for 10 min at 43, 45, 47, 49, 51, 53, 55, or 100 °C and the first electrical conductance (EC_1_) was measured using an EC meter (CHS30, Mettler Toledo Inc., Greifensee, Switzerland). The peel discs in the sucrose solution were then autoclaved at 121 °C for 20 min, cooled, and then used for measuring EC_2_. The EL was calculated as follows:

EL = (EC_1_/EC_2_) EC × 100 (5)

where EC_1_ and EC_2_ are the first electrical conductance and second electrical conductance after autoclaving at 121 °C, respectively.

The percentage of injury was calculated according to the method described by Lim et al. [54], with the following equation:

% injury = %EL_(t)_ − %EL_(c)_/100 − %EL_(c)_ × 100 (6)

where %EL_(t)_ and %EL_(c)_ are the EL percentages for the heat-treated and control (unheated) peel discs, respectively. Peel discs treated at 100 °C represented 100% heat injury. The injury percentages were transformed as follows:

% adjusted injury = (%injury_(t)_/%injury_(100__°C)_) × 100 (7)

where, % injury_(t)_ and % injury_(100__°C)_ are the injury percentages for the heat-treated and control (unheated) peel discs, respectively.

### 4.9. Statistical Analysis

The data were expressed as mean ± SE. The mean values of the treatment were separated using Duncan’s multiple range test after analysis of variance at *p* < 0.05. All the experiments were performed at least in triplicates for each treatment. In addition, the analyses of data for fruit properties and free sugar and organic acid contents were performed using Student’s *t*-test. The statistical programs used were R version 3.6.3 software package R Studio, Boston, MA, USA) and PASW version 18 (SPSS Inc., Chicago, IL, USA). 

## Figures and Tables

**Table 1:** Flavonoid contents (mg/100 g 70% ethanol dry extract) according to the degree of sunburn in Satsuma mandarin fruits.

Sample	Stage	Rutin	Narirutin	Naringin	Hesperidin	Neohesperidin	Nobiletin	Tangeretin	Total
Pulp	Control	99.6 ± 6.4 d	1034.6 ± 73.7 e	ND ^a^	1170.6 ± 48.3 d	ND	ND	ND	2304.8 ± 122.5 d
	I	128.8 ± 11.8 c	1319.2 ± 24.4 d	ND	1215.0 ± 68.0 cd	ND	ND	ND	2663.1 ± 92.0 c
	II	163.7 ± 3.6 b	1638.8 ± 33.1 b	ND	1423.9 ± 20.9 bc	ND	ND	ND	3226.4 ± 39.1 b
	III	197.3 ± 1.3 a	1850.4 ± 25.2 a	ND	1584.1 ± 110.7 ab	ND	ND	ND	3631.9 ± 114.4 a
	IV	157.3 ± 2.4 b	1501.7 ± 31.4 c	ND	1683.3 ± 74.0 a	ND	ND	ND	3342.3 ± 102.9 ab
Peel	Control	587.2 ± 51.9 e	3134.4 ± 146.1 ab	34.3 ± 2.0 a	4615.1 ± 166.6 ab	Traces	116.5 ± 2.6 a	48.9 ± 0.3 a	8536.3 ± 345.9 b
	I	721.4 ± 7.5 d	2747.3 ± 64.0 b	24.6 ± 3.4 ab	3968.1 ± 288.0 b	Traces	94.6 ± 4.1 b	39.3 ± 1.2 b	7595.3 ± 335.5 bc
	II	1098.6 ± 26.2 b	2830.3 ± 50.1 b	23.2 ± 0.3 ab	4386.2 ± 204.7 ab	Traces	81.3 ± 1.7 c	33.0 ± 0.6 c	8452.6 ± 269.5 bc
	III	1211.0 ± 39.3 a	3469.0 ± 166.1 ab	12.3 ± 6.2 bc	4803.3 ± 214.8 a	Traces	107.5 ± 2.3 a	41.7 ± 1.0 b	9644.9 ± 420.3 a
	IV	980.4 ± 28.5 c	2016.1 ± 175.7 c	6.4 ± 6.4 c	4297.0 ± 295.6 ab	Traces	64.7 ± 4.6 d	23.0 ± 1.6 d	7387.6 ± 241.1 c

The values represent the mean ± standard error (SE) of three replicates. The means followed by different alphabets within columns are significantly different according to Duncan’s multiple range test (*p* < 0.05). ^a^ ND, not detected.

**Table 2:** Physicochemical analysis of sunburned and non-sunburned fruits of *Citrus unshiu* ‘Nichinan No. 1’.

Treatment	TSS	Acidity (%)	Firmness	L*	a*	b*
Control	9.0 ± 0.1 ***	1.2 ± 0.2 ***	8.0 ± 0.2 ***	69.6 ± 0.2 ***	18.4 ± 0.5	70.9 ± 0.3 ***
Sunburn	8.6 ± 0.1	1.1 ± 0.2	12.5 ± 0.6	62.6 ± 0.8	16.8 ± 0.9	60.8 ± 1.4

The values represent mean ± SE of 30 replicates. *** *p* < 0.001 (Student’s *t*-test). Harvest time: 19 October 2020.

## Data Availability

Not applicable.

## References

[1] Rabinowitch H.D., Kedar N., Budowski P. (1974). Induction of sunscald damage in tomatoes under natural and controlled conditions. Sci. Hortic..

[2] Parchomchuk P., Meheriuk M. (1996). Orchard cooling with pulsed over- tree irrigation to prevent solar injury and improve fruit quality of ‘Jonagold’ apples. Hortscience.

[3] Schrader L.E., Zhang J., Duplaga W.K. (2001). Two types of sunburn in apple caused by high fruit surface (peel) temperature. Plant Health Prog..

[4] Tarara J.M., Spayd S.D. (2005). Tackling ‘sunburn’ in red wine grapes through temperature and sunlight exposure. Good Fruit Grow..

[5] Racsko J., Schrader L.E. (2012). Sunburn of apple fruit: Historical background, recent advances and future perspectives. Crit. Rev. Plant Sci..

[6] Masson-Delmotte V., Zhai P., Pirani A., Connors S.L., Péan C., Berger S., Caud N., Chen Y., Goldfarb L., Gomis M.I., IPCC (2021). Climate Change: The Physical science basis. Contribution of Working Groups I to the sixth Assessment Report of the Intergovernmental Panel on Climate Change.

[7] Korea Meteorological Administration Korean Climate Change Assessment Report. http://www.climate.go.kr/home/cc_data/2020/Korean_Climate_Change_Assessment_Report_2020_1_summary.pdf.

[8] Park Y., Kim M., Yun S.K., Kim S.S., Joa J. (2022). A simple model for predicting sunburn on Satsuma mandarin fruit. Sci. Hortic..

[9] Benavente-García O., Castillo J., Marin F.R., Ortuño A., Del Río J.A. (1997). Uses and properties of citrus flavonoids. J. Agric. Food Chem..

[10] Bocco A., Cuvelier M., Richard H., Berset C. (1998). Antioxidant activity and phenolic composition of citrus peel and seed extracts. J. Agric. Food Chem..

[11] Aschoff J.K., Kaufmann S., Kalkan O., Neidhart S., Carle R., Schweiggert R.M. (2015). In vitro bioaccessibility of carotenoids, flavonoids, and vitamin C from differently processed oranges and oranges juices [*Citrus sinensis* (L.) Osbeck]. J. Agric. Food Chem..

[12] Laganá V., Giuffrè A.M., De Bruno A., Poiana M. (2022). Formulation of biscuits fortified with a flour obtained from bergamot by-products (*Citrus bergamia*, Risso). Foods.

[13] Kim S.S., Park K.J., Yun S.H., Choi Y.H. (2019). Bioactive compounds and antioxidant capacity of domestic citrus cultivar ‘Haryejosaeng’. Korean J. Food Preserv..

[14] Jeju Special Self-Governing Province Citrus Marketing & Shipping Association Citrus Annual Distribution Processing Analysis. http://www.citrus.go.kr.

[15] Minessy F.A., Nasr T.A.A., El-Shurafa M.Y. Citrus fruit temperature in relation to sunburn. Proceedings of the Conference on Tropical and Subtropical Fruits.

[16] Wan J.F., Li J., Chen J.Z. (2012). Changes in antioxidant metabolism in the fruit pericarps of citrus during sunburn development. Acta Hortic. Sin..

[17] Kang S.-B., Moon Y.-E., Yankg K.-R., Joa J.-H., Lee H.J. (2019). Effect of the harvest season on the yield and growth of unripe fruit and biennial flowering of ‘Miyagawa’ satsuma mandarin in open field cultivation. Korean J. Environ. Agric..

[18] Tasi M.-S., Lee T.-C., Chang P.-T. (2013). Comparison of paper bags, calcium carbonate, and shade nets for sunscald protection in ‘Murcott’ Tangor fruit. HortTechnology.

[19] Lee T.-C., Zhong P.-J., Chang P.-T. (2015). The effects of preharvest shading and postharvest storage temperatures on the quality of ‘Ponkan’ (*Citrus reticulate* Blanco) mandarin fruits. Sci. Hortic..

[20] Ennab H.A., El-Sayed S.A., Abo El-Enin M.M.S. (2017). Effect of kaolin applications on fruit sunburn, yield and fruit quality of balady mandarin (*Citrus reticulate*, Blanco). Menoufia J. Plant Prod..

[21] Tajvar Y., Ghazvini R.F., Hamidoghli Y., Sajedi R.H. (2011). Antioxidant changes of Thomson navel orange (*Citrus sinensis*) on three rootstocks under low temperature stress. Hortic. Environ. Biotechnol..

[22] Kim M., Yun S.K., Kim S.S., Park Y., Joa J., Han S. (2021). Influence of freezing temperatures on metabolite composition and antioxidant activity in Shiranuhi mandarin. Sci. Horti..

[23] Wang L.-J., Loescher W., Duan W., Li W.-D., Yang S.-H., Li S.-H. (2009). Heat acclimation induced acquired heat tolerance and cross adaptation in different grape cultivars: Relationships to photosynthetic energy partitioning. Funct. Plant Biol..

[24] Araújo M., Santos C., Dias M.C., Alves F., Filho W.L., Azeiteiro U. (2018). Can Young olive plants overcome heat shock?. Theory and Practice of Climate Adaptation.

[25] Yasuhiko K., Tomoka S., Mari N. (2020). Studies on the mechanism of sunscald occurrence and its mitigation in citrus fruit. Bull. Yamaguchi Agric. Fore. Gene Tec. Ctr..

[26] Tripoli E., La Guardia M., Giammanco S., Di Majo D., Giammanco M. (2007). Citrus flavonoids: Molecular structure, biological activity and nutritional properties: A review. Food Chem..

[27] Giuffrè A.M., Zappia C., Capocasale M. (2017). Physico-chemical stability of blood orange juice during frozen storage. Int. J. Food Prop..

[28] Giuffrè A.M. (2019). Bergamot (*Citrus bergamia*, Risso): The effects of cultivar and harvest date on functional properties of juice and cloudy juice. Antioxidants.

[29] Sayyari M., Ghanbari F., Fatahi S., Bavandpour F. (2013). Chilling tolerance improving of watermelon seedling by salicylic acid seed and foliar application. Not. Sci. Biol..

[30] Petracek P.D. (1997). Peel morphology and fruit blemishes. Citrus Flowering and Fruiting Short Course.

[31] Wang J., Hao H., Liu R., Ma Q., Xu J., Chen F., Cheng Y., Deng X. (2014). Comparative analysis of surface wax in mature fruits between satsuma mandarin (*Citrus unshiu*) and ‘Newhall’ navel orange (*Citrus sinensis*) from the perspective of crystal morphology, chemical composition and key gene expression. Food Chem..

[32] Bain J.M. (1958). Morphological, anatomical, and physiological changes in the developing fruit of the Valencia orange, *Citrus sinesis* (L.) Osbeck. Aust. J. Bot..

[33] Spiegel-Roy P., Goldschmidt E.E. (1996). Biology of Citrus Cambridge.

[34] Schrader L.E., Kahn C.B., Felicetti D.A., Sun J., Xu J., Zhang J. Effects of high temperature and high solar irradiance on sunburn, quality, and skin pigments of apple fruit. Proceedings of the X International Symposium on Integrating Canopy, Rootstock and Environmental Physiology in Orchard Systems.

[35] Hörtensteiner S. (2006). Chlorophyll degradation during senescence. Annu. Rev. Plant Biol..

[36] Wang Q.-L., Chen J.-H., He N.-Y., Guo F.-Q. (2018). Metabolic reprogramming in chloroplasts under heat stress in plants. Int. J. Mol. Sci..

[37] Hu S., Ding Y., Zhu C. (2020). Sensitivity and responses of chloroplast to heat stress in plants. Front. Plant Sci..

[38] Chen L.-S., Li P., Cheng L. (2008). Effects of high temperature coupled with high light on the balance between photooxidation and photoprotection in the sun-exposed peel of apple. Planta.

[39] Felicetti D.A., Schrader L.E. (2008). Changes in pigment concentrations associated with the degree of sunburn browning of ‘Fuji’ apple. J. Am. Soc. Hort. Sci..

[40] Felicetti D.A., Schrader L.E. (2009). Changes in pigment concentrations associated with sunburn browning of five apple cultivars. I. Chlorophylls and carotenoids. Plant Sci..

[41] Sdiri S., Bermejo A., Aleza P., Navarro P., Salvador A. (2012). Phenolic composition, organic acids, sugars, vitamin C and antioxidant activity in the juice of two new triploid late-season mandarins. Food Res. Int..

[42] Kim M., Yun S.K., Kim S.S., Park Y., Joa J., Han S., Shin K., Song K.J. (2021). Response of citrus to freezing tolerance differs depending on genotypes and growing conditions. Hortic. Environ. Biotechnol..

[43] Song E.-Y., Choi Y.-H., Kang K.-H., Koh J.-S. (1998). Free sugar, organic acid, hesperidin, naringin and inorganic elements changes of Cheju citrus fruits according to harvest. Korean J. Food Sci. Technol..

[44] Yamazaki Y., Suh D.-Y., Sitthithaworn W., Ishiguro K., Kobayashi Y., Shibuya M., Ebizuka Y., Sankawa U. (2001). Diverse chalcone synthase superfamily enzymes from the most primitive vascular plant, *Psilotum nudum*. Planta.

[45] Takahashi A., Ohnishi T. (2004). The significance of the study about the biological effects of solar ultraviolet radiation using the exposed facility on the international space station. Biol. Sci. Space.

[46] Rapisarda P., Bianco M.L., Pannuzzo P., Timpanaro N. (2008). Effect of cold storage on vitamin C, phenolics and antioxidant activity of five orange genotypes [*Citrus sinensis* (L.) Osbeck]. Postharvest Biol. Technol..

[47] Roussos P.A. (2011). Phytochemicals and antioxidant capacity of orange (*Citrus sinensis* (L.) Osbeck cv. Salustiana) juice produced under organic and integrated farming system in Greece. Sci. Hortic..

[48] Lim P.O., Kim H.J., Nam H.G. (2007). Leaf Senescence. Annu. Rev. Plant Biol..

[49] Felicetti D.A., Schrader L.E. (2008). Photooxidative sunburn of apples: Characterization of a third type of apple sunburn. Int. J. Fruit Sci..

[50] Clément C., Burrus M., Audran J.-C. (1996). Floral organ growth and carbohydrate content during pollen development in *Lilium*. Amer. J. Bot..

[51] Lichtenthaler H.K. (1987). Chlorophylls and carotenoids: Pigments of photosynthetic biomembranes. Meth. Enzymol..

[52] Jakhar S., Mukherjee D. (2014). Chloroplast pigments, proteins, lipid peroxidation and activities of antioxidative enzymes during maturation and senescence of leaves and reproductive organs of *Cajanus cajan* L.. Physiol. Mol. Biol. Plants..

[53] Dionisio-Sese M.L., Tobita S. (1998). Antioxidant responses of rice seedlings to salinity stress. Plant Sci..

[54] Lim C.C., Arora R., Townsend E.C. (1998). Comparing Gompertz and Richards functions to estimate freezing injury in Rhododendron using electrolyte leakage. J. Am. Soc. Hortic. Sci..

